# Evaluation of a resistance training program for adults with or at risk of developing diabetes: an effectiveness study in a community setting

**DOI:** 10.1186/1479-5868-8-50

**Published:** 2011-05-25

**Authors:** Karl E Minges, Glen Cormick, Edna Unglik, David W Dunstan

**Affiliations:** 1 Baker IDI Heart and Diabetes Institute, Melbourne, Victoria, Australia; 2 The University of Queensland, School of Population Health, Cancer Prevention Research Centre, Brisbane, Australia; 3 School of Exercise and Nutrition Sciences, Deakin University, Melbourne, Australia; 4 ECU Health and Wellness Institute, Edith Cowan University, Perth, Australia; 5 Department of Epidemiology and Preventive Medicine, Monash University, Melbourne, Australia

## Abstract

**Background:**

To examine the effects of a community-based resistance training program (Lift for Life^®^) on waist circumference and functional measures in adults with or at risk of developing type 2 diabetes.

**Methods:**

Lift for Life is a research-to-practice initiative designed to disseminate an evidence-based resistance training program for adults with or at risk of developing type 2 diabetes to existing health and fitness facilities in the Australian community. A retrospective assessment was undertaken on 86 participants who had accessed the program within 4 active providers in Melbourne, Australia. The primary goal of this longitudinal study was to assess the effectiveness of a community-based resistance training program, thereby precluding a randomized, controlled study design. Waist circumference, lower body (chair sit-to-stand) and upper body (arm curl test) strength, and agility (timed up-and-go) measures were collected at baseline and repeated at 2 months (n = 86) and again at 6 months (n = 32).

**Results:**

Relative to baseline, there was a significant decrease in mean waist circumference (-1.9 cm, 95% CI: -2.8 to -1.0) and the timed agility test (-0.8 secs, 95% CI: -1.0 to -0.6); and significant increases in lower body (number of repetitions: 2.2, 95% CI: 1.4-3.0) and upper body (number of repetitions: 3.8, 95% CI: 3.0-4.6) strength at the completion of 8 weeks. Significant differences remained at the 16 week assessment. Pooled time series regression analyses adjusted for age and sex in the 32 participants who had complete measures at baseline and 24-week follow-up revealed significant time effects for waist circumference and functional measures, with the greatest change from baseline observed at the 24-week assessment.

**Conclusions:**

These findings indicate that an evidence-based resistance training program administered in the community setting for those with or at risk of developing type 2 diabetes, can lead to favorable health benefits, including reductions in central obesity and improved physical function.

## Background

Exercise, along with diet and medication, plays an important role in the management of type 2 diabetes. Resistance exercise may provide unique health and fitness benefits for the treatment of a number of chronic diseases [1,2]. Specifically, several studies have now demonstrated that supervised resistance training may be a viable and effective exercise modality for the improvement of glycemic control in middle-aged and older adults with type 2 diabetes [3-5]. The American College of Sports Medicine and the American Diabetes Association now specifically recommends the use of resistance training, in addition to aerobic exercise, to help enhance and maintain muscular strength and endurance, maintain lean muscle mass and aiding in the management of glycemic control [6].

Typically, the positive benefits of resistance training have been observed using programs that have involved supervised exercise sessions in well-controlled laboratory, clinical or gymnasium settings. An advantage of this approach is that exercise prescription can be carefully monitored to encourage both appropriate adherence and exercise progression to stimulate metabolic changes. However, from a public health perspective, it is important to understand the effectiveness of maintenance programs undertaken in the community setting. While maintenance programs conducted in the home can provide convenience and flexibility [7], we have previously reported that home-based resistance training for 6 months was not effective for maintaining the improvements in glycemic control associated with 6 months of supervised training in older persons with type 2 diabetes [8]. The apparent ineffectiveness of home-based training was most likely due to reduced adherence, the absence of ongoing supervision, and decreased exercise training volume and intensity since the hand and leg weights used in the home could not replicate the workloads experienced in the supervised setting.

Training programs in community facilities such as health and fitness centers or gymnasiums offer greater access to resistance exercise equipment, supervision and group interaction than home-based training. Such training attributes reflect several of the key social and environmental factors that can beneficially influence the maintenance of physical activity behaviors [9,10]. In a recent randomized controlled trial, we demonstrated that a 12-month resistance training program undertaken in a community-based health and fitness center was feasible and more effective than a control program (where participants received one dumbbell for use during the intervention) for improving muscle strength and blood glucose control in adults with type 2 diabetes [11]. A recent review has highlighted gaps in the literature, particularly the absence of viable resistance training options that offer practical, sustainable or economical strategies for physical activity in those with type 2 diabetes [2].

In 2005, a major research-to-practice initiative was undertaken that included the development of a community-based resistance training program titled Lift for Life for people with or at risk of developing type 2 diabetes (http://www.liftforlife.com.au). The goal was to translate the evidence-based resistance training program to existing community health and fitness facilities through the development of a network of accredited providers in Australian cities.

Here, we report the findings from a cross-sectional assessment of older, and overweight or obese participants with or at risk of developing type 2 diabetes who have accessed the resistance training program within five active providers in metropolitan Melbourne, during 35 consecutive months. Specifically, we have examined the effects of the Lift for Life program on functional and adiposity measures.

## Methods

### Design of Study

An audit of the five providers (community health and fitness centres) in Melbourne, Victoria who had received accreditation to implement the Lift for Life program, and who had been an active provider for at least six months, was undertaken in February 2006. The audit encompassed the collation of data that had been collected for each participant in the respective facilities post-accreditation. This involved the photocopying of assessment forms for each participant who had received an initial assessment prior to commencing the program. During the audit process, it was identified that one provider had not complied with the intended assessment procedures and hence this provider was not included in the analysis. For the remaining four providers, the following information was collected for each participant: demographics (age, sex), waist circumference, upper body strength (arm curl test), agility (timed up-and-go) and lower body strength (chair sit-to-stand). For each participant the initial baseline assessment and the most recent assessment were obtained.

### Accreditation

As a requirement of the accreditation process, all Lift for Life trainers attend a 2-day training workshop. Within this training, demonstrations of the testing protocols are reviewed along with the provision of written instructions. All trainers are required to provide a satisfactory level of competency with the testing protocols, as determined by a final written assessment.

### Lift for Life resistance training intervention

**Figure 1:**
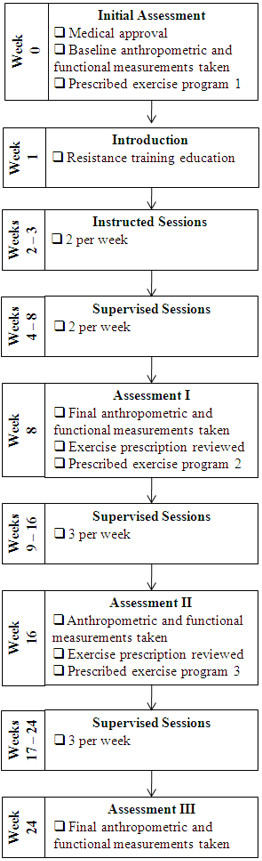
**Stages of the Lift for Life program**.

Lift for Life is a progressive resistance training program structured over three phases of approximately eight weeks each (See Figure 1). In accordance with current resistance exercise guidelines for people with and without type 2 diabetes [12,13] the program uses a similar resistance training protocol as the clinical trials [3,11]. Lift for Life incorporates isotonic resistance training equipment (pin weighted machines and free weights) with an emphasis on continual progressive overload (increments of 2-10%). Participants attended exercise sessions in small groups of 8-12 people under the supervision of exercise trainers (Physiotherapists, Exercise Physiologists and experienced Certificate IV Personal Trainers) who have received specialized training, as part of the Lift for Life accredited process. Before commencement of the resistance training program, participants must obtain approval from a medical practitioner and undergo a baseline assessment conducted by the program provider. Participants are then prescribed a customised resistance training program and are gradually introduced to the program throughout the first 8-week phase. This phase requires participants to undertake two sessions per week, at which point the first phase finishes and another assessment is undertaken.

The participant's exercise prescription is then reviewed and a new customised program implemented before the participant commences the second 8-week phase; but with the goal of completing three sessions per week and ending with another assessment at week 16. The final phase is a replication of the previous phase, and if warranted, minor exercise prescription adjustments are made. Achievement of the entire program would result in a total of 24 weeks of resistance training and approximately 64 group sessions.

The cost of each session per participant varies among Lift for Life providers, and is approximately $10 - $15 AUD. Some private health insurers subsidise the cost of the program.

### Subjects

A convenience sample was obtained from subjects who had participated in the program at their chosen local facility within the duration of 35 consecutive months, from February 2006 to December 2008.

Participants with severe orthopedic, cardiovascular or respiratory conditions that would preclude participation in an exercise program, or those with a medical condition listed in the American College of Sports Medicine (ACSM) absolute exercise contraindications [14] were not granted permission by their doctor to participate and thus were also excluded. The study was approved by the International Diabetes Institute Ethics Committee, and written informed consent was obtained from all participants. Overall, 86 participant were recruited for the study.

### Testing Procedures

#### Anthropometry and Body Composition

Existing equipment located in the respective facility was used to ascertain anthropometric measures. Within the Lift for Life procedures, height (cm) was measured using a stadiometer and body weight (kg) was assessed using calibrated scales to the nearest 0.1 kg. Waist circumference was measured using a non-elastic measuring tape at the mid-point between the lower border of the ribcage and the iliac crest. All of the data were collected by the program providers.

#### Functional measures

A validated battery of three physical test items [15], developed specifically for older adults, was used to assess aspects of functional fitness. These tests included: chair sit-to-stand (number of complete movements undertaken in 30 seconds; lower body strength), arm curl (number of complete movements undertaken using a standard dumbbell weight in 30 seconds; upper body strength) and timed up-and-go (agility course over 8 feet - time taken to complete).

### Statistical Analysis

Statistical analysis was conducted using Stata version 10.0 (STATA, College Station, TX, USA). The net differences were calculated by subtracting the changes from baseline for each time point (week 8, week 16, and week 24 assessments). Descriptive data at baseline was analyzed using paired t-tests. The time effects for anthropometric and functional variables were examined using pooled time series regression analysis for longitudinal data with random effects models. Missing baseline data were replaced with the mean measurement for the respective variable for the total group: waist circumference (1 imputation), chair sit-to-stand (1 imputation), arm curl test (2 imputations) and agility - up-and-go test (3 imputations).

## Results

### Subject characteristics

**Figure 2:**
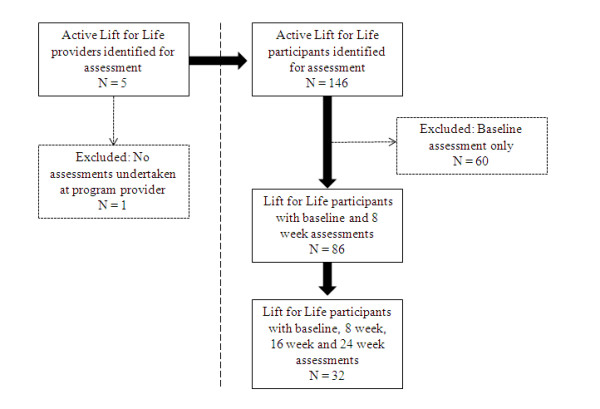
**Schematic overview of the assessment**.

At the time the study was conducted, 146 active Lift for Life participants were identified from the four Lift for Life providers (Figure 2). Sixty of these participants had completed the baseline assessment but did not have any follow-up data and hence were excluded from the analyses. Thus, 86 participants had completed both the baseline and week 8 assessments and were included in the analysis. Of these, 32 had completed all four testing time points (baseline, week 8, week 16 and week 24) (see Table 1 for summary characteristics). For those completing the first assessment at week 8, this yielded a compliance of 37% for completion of the entire program. There were no significant differences in terms of age, sex or baseline waist circumference between the 54 subjects who only completed the first assessment (week 8) and the 32 subjects who completed the entire program (week 24). Significant between-group differences were noted and observed to be higher for the subjects who completed the entire program for the baseline arm curl test (upper body) and the baseline chair sit-to-stand test (lower body), but there was no significant association for between-group differences for the baseline up-and-go test. Furthermore, no significant between-group differences were observed for any of the functional measures for between-group comparisons at week 8.

**Table 1:** Baseline characteristics of the Lift for Life participants

Participants with Baseline and 8-week Assessments	
*n*	86
Age (years)	66.4 ± 8.7 Range: 45 - 93
Gender (M/F)	27/59
	
**Participants with Baseline, 8-week, 16-week and 24-week Assessments**	
*N*	32
Age (yrs)	65.1 ± 7.5 Range: 45 - 75
Gender (M/F)	8/24

Data are n or mean ± SD

### Changes in anthropometric and functional measurements

#### Baseline to 8-week Assessment

The mean changes in waist circumference, lower body strength (chair sit-to-stand), upper body strength (arm curl test) and timed agility test measurements from baseline to the week 8 assessment for the 86 participants are shown in Table 2. Significant decreases in both waist circumference (-1.9 cm; 95% CI: -2.8 to -1.0) and the timed up-and-go test (-0.8 seconds; 95% CI: -1.0 to -0.6); and significant increases in chair sit-to-stand (2.2 repetitions; 95% CI: 1.4 to 3.0) and arm curl test (3.8 repetitions; 95% CI: 3.0 to 4.6) was observed at the completion of the week 8 assessment.

**Table 2:** Changes in anthropometric and functional variables from baseline to the completion of the 8-week phase (*n *= 86).

	Baseline Mean (SD)	Week 8 Mean (SD)	Adjusted* mean difference from baseline to 8-week assessment (95% CI)
Waist circumference (cm)	104.8 (14.9)	102.9 (15.2)	-1.9 (-2.8 to -1.0) ^§^
Chair sit-to-stand (n)	13.1 (3.9)	15.4 (4.6)	2.2 (1.4 to 3.0) ^§^
Arm curl (n)	16.4 (4.4)	20.2 (4.1)	3.8 (3.0 to 4.6) ^§^
Timed up-and-go (secs)	6.5 (1.7)	5.7 (1.6)	-0.8 (-1.0 to -0.6) ^§^

*Adjusted for age and sex

^§ ^P-value for time trend < 0.0001

#### Baseline to Final Assessment (24-weeks)

Table 3 presents the changes in anthropometric and functional variables from baseline to the final assessment at week 24, for the 32 participants in which these measurements were available. The pooled time series regression analysis adjusted for age and sex revealed significant time effects for all variables: decreases in waist circumference (-4.9 cm; 95% CI: -6.7 to -3.0) and timed agility test (-1.2 seconds; 95% CI: -1.5 to -0.9); and increases in lower body (4.2 repetitions; 95% CI: 2.9 to 5.6) and upper body (5.3 repetitions; 95% CI: 4.0 to 6.6) strength. For all variables, larger changes were observed at the week 24 assessment relative to other assessment time points. These results do not significantly differ if the baseline data imputations for waist circumference, chair sit-to-stand, arm curl test and agility were not applied.

**Table 3:** Changes in anthropometric and functional variables from baseline to final assessment (24-weeks) (*n *= 32).

		Adjusted* mean difference from baseline		
		
	Baseline Mean (SD)	Week 8 Mean (95% CI)	Week 16 Mean (95% CI)	Week 24 Mean (95% CI)
Waist circumference (cm)	105.7 (15.6)	-2.3 (-4.2 to -0.4)	-2.5 (-4.4 to -0.6)	-4.9 (-6.7 to -3.0) ^§^
Chair sit-to-stand (n)	14.3 (4.2)	1.6 (0.2 to 3.0)	3.1 (1.7 to 4.5)	4.2 (2.9 to 5.6) ^§^
Arm curl (n)	17.7 (4.9)	2.6 (1.3 to 3.9)	5.1 (3.8 to 6.4)	5.3 (4.0 to 6.6) ^§^
Timed up-and-go (secs)	6.4 (1.6)	-0.8 (-1.1 to -0.4)	-1.0 (-1.4 to -0.7)	-1.2 (-1.5 to -0.9) ^§^

*Adjusted for age and sex

^§ ^P-value for time trend < 0.0001

## Discussion

The results of this study indicate that the community-based Lift for Life resistance training program was effective for improving waist circumference, lower and upper body strength and agility in adults with or at risk of developing type 2 diabetes. Greater improvements in these anthropometric and functional measurements were seen at the completion of the full 24-week program relative to other testing time points. The findings support the effectiveness of the Lift for Life program for improving health and physical function and reinforce the importance of encouraging individuals to adhere to the full 24-week program to maximize their gains.

Previous studies conducted in well-controlled exercise testing laboratories have demonstrated significant reductions in central obesity (waist circumference) following resistance training in individuals with or at risk of type 2 diabetes [16-18]. The 4.9 cm reduction in waist circumference observed following the completion of the Lift for Life program compares favourably with the results from our previous randomized controlled trial [3] that investigated the effects of the combination of a modest weight loss diet and a similar resistance training program in older adults with type 2 diabetes. In that study, a 6.9 cm decrease was observed in waist circumference compared to baseline measurements, which coincided with a significant 1.2% reduction in glycated haemoglobin (HbA1c) [3]. While glycemic control was not assessed in the present study, the relationship between central obesity, glycemic control and resistance training is well-documented [19] and it could be speculated that the reduction in waist circumference observed in the Lift for Life participants may have also favoured improved glycemic control during this period.

Similarly, the improvement in upper body and lower body strength is consistent with previous investigations involving resistance training in this population [20]. The 30% improvement in upper body and lower body strength seen from baseline to 24 weeks, along with the 19% improvement in agility suggests that the Lift for Life program can have a meaningful impact on physical function in older adults with or at risk of developing type 2 diabetes. This is an important consideration since adults with type 2 diabetes have an increased susceptibility to declines in physical function and muscle strength compared to non-diabetic individuals, which invariably impacts on physical function and well-being [4]. Whilst baseline upper body and lower body strength values were higher in those who completed the entire program, these differences were no longer evident at the week 8 measures. This finding could have led us to speculate that having higher strength levels at the start of the program may have influenced participation levels longer term, yet, with the absence of qualitative data relating to reasons for not continuing, it is not possible to draw definitive conclusions since there may have been a number of contributors to ceasing participation in this group.

Lift for Life is an example of research translation to the wider community, an important step that is rarely achieved within scientific research. The implementation of an evidence-based program in the Australian community setting is timely given the increased recognition of preventative health care services within public health and government. Such an undertaking requires extensive collaboration between the scientists and the practitioners who are experienced in delivering exercise programs in the community. Invariably, considerable time is required to establish and foster these collaborative links, as evidenced by the fact that the Lift for Life program has been under development for approximately seven years.

An increased emphasis on the establishment of community-based resistance training programs for older Australians with and without diabetes, such as Lift for Life, is clearly warranted [2]. While national prevalence figures relating to resistance training participation is lacking, it has been reported in a small study of regional Australians that the overall prevalence of participation in gym-based resistance training is poor, with less than 14% of the overall sample engaged in strength developing activities [21]. Alarmingly, the poorest participation rates are evident in the older adults, with 7% of adults age 55 or greater participating in resistance training [21]. Low participation rates have also been observed in older adults in a recent Australian Bureau of Statistics survey of recreational activity [22]. Notably, older adults are a population whom it can be expected to derive the greatest benefits from resistance training, since it is well documented that advancing age coincides with substantial losses in muscle mass, which invariably impacts on physical function and well-being [19]. As such, continued efforts should be made to increase awareness of the need for and actual participation in evidence-based resistance training programs in older adults, particularly those in the later stages of life. Furthermore, these efforts should include consideration from policymakers to subsidise or at worst, part-subsidise the cost of such exercise programs, since out-of-pocket expense may deter people from initiating the program. This may be particularly beneficial for older adults, given possible financial limitations.

Interpretation of the study findings is limited by the lack of a randomized controlled study design, an approach that is difficult to achieve in community-based programs that are focused on dissemination rather than scientific research; therefore, the true effect of the intervention is difficult to ascertain. Furthermore, in contrast to the controlled scientific setting, in the 'real-life' community environment there is an increased exposure to having missing data points since data collection was contingent on the instructors adhering to the Lift for Life requirements. Additionally, the assessments at each time point may have been undertaken by different instructors and therefore could have affected the precision of the measurements.

Our primary intention for the current study was to provide a snapshot of those participants who had engaged in the Lift for Life program in the metropolitan Melbourne providers. This approach is limited by the timing of each individuals' involvement in the program, as indicated by the fact that substantially less numbers were available for the assessment of the full program (24 weeks) compared to a shorter time frame (8 weeks). Furthermore, given that this evaluation is limited by a convenience sample of providers in one urban location, the results may not be wholly reflective to all Australians, and are more likely to represent urban populations. To enhance generalizability of community-based findings, future studies should seek equal gender representation and address additional socio-demographic characteristics (ethnicity, education, income, occupational status, household composition, marital status and number of comorbidities).

## Conclusions

With the ageing population and the rising epidemics of type 2 diabetes and obesity, it is vital to support and maintain health-enhancing physical activity in the community setting [2]. We have demonstrated that the community-based Lift for Life program, an evidence-based resistance training program specifically developed for those with or at risk of developing type 2 diabetes, can lead to favourable health benefits, including reductions in central obesity and improved physical function. The ongoing establishment of Lift for Life providers throughout the Australian community will provide more widespread access to evidence-based physical activity in older adults.

## Competing interests

DWD (as the Lift for Life creator) receives a proportion of the annual payment that Baker IDI attracts from Fitness Australia under its Lift for Life licensing arrangements.

## Authors' contributions

KEM performed statistical analyses, contributed to study design and helped to draft the manuscript. GC participated in the design of the study and data acquisition. EU participated in the design of the study and data acquisition. DWD conceived the study, participated in its design, performed statistical analyses and helped to draft the manuscript. All authors read and approved the final manuscript.
